# Screen time is negatively associated with sleep quality and duration only in insufficiently active adolescents: A Brazilian cross-sectional school-based study

**DOI:** 10.1016/j.pmedr.2023.102579

**Published:** 2023-12-25

**Authors:** Amanda B. dos Santos, Wagner L. Prado, William R. Tebar, Jared Ingles, Gerson Ferrari, Priscila K. Morelhão, Luan O. Borges, Raphael M. Ritti Dias, Victor S. Beretta, Diego G.D. Christofaro

**Affiliations:** a São Paulo State University (Unesp), School of Technology and Sciences, Presidente Prudente, SP, Brazil; b California State University-San Bernardino, San Bernardino, USA; c Faculty of Health Sciences, Universidad Autónoma de Chile, Providencia 7500912, Chile; d Universidade Federal de São Paulo (UNIFESP), São Paulo, Brazil; e Universidade Nove de Julho, São Paulo, Brazil

**Keywords:** Sedentary behavior, Sleep quality, Television, Exercise, Youth

## Abstract

**Background:**

Time spent on screen devices affects sleep quality and duration leading to several health impairments in youth. Although physical activity (PA) benefits sleep patterns and decreases screen time in adolescents, it is unclear whether the relationship between sleep quality/duration and screen time could be influenced by PA levels.

**Objective:**

To analyze the association between sleep quality and duration with screen time in Brazilian adolescents according PA levels.

**Methods:**

The sample included 1010 adolescents aged 13.2 ± 2.4 years (n = 556 females − 55 % of the sample). Sleep quality and sleep duration, and PA were assessed by Mini Sleep and Baecke questionnaires, respectively. Participants in the highest quartile were classified as physically active. Screen time was analyzed by the self-reported number of hours spent on different screen devices (i.e., television, computer, videogame, and cellphone/tablet). Participants in the highest tertile were classified as having high screen time. Sex, age, and body mass index were considered covariates in binary logistic regression models.

**Results:**

Poor sleep quality was observed in 52.3 % of the sample, whereas 46.6 % reported sleeping less than eight hours/day. High screen time was associated with poor sleep quality (OR = 1.45; 95 %CI = 1.01–2.12) and insufficient sleep duration (OR = 1.52; 95 %CI = 1.01–2.03) in adolescents insufficiently active. There were no associations between screen time and sleep parameters in active adolescents.

**Conclusion:**

High screen time was associated with poor sleep quality and insufficient sleep duration only in insufficiently active adolescents. These results suggest that high PA levels may contribute to improving sleep patterns in pediatric population.

## 1 Introduction

Technological advancements of the last century have led to a notable increase in sedentary behavior. Long-term engagement in screen activities (e.g., watching Tv, using computers, smartphones, and tablets) contributes to sedentary behavior (Schaan et al., 2019, Trott et al., 2022). The time spent on screen devices has been associated with impaired sleep parameters in adolescents (Felden et al., 2016), such as a shorter duration of sleep (Sanz-Martín et al., 2022, Twenge et al., 2019). Given the elevated prevalence of excessive time in screen devices in adolescents (i.e., ∼71 %) (Schaan et al., 2019), strategies focused on limiting screen time are necessary in this population.

Sleep quality and deprivation have been associated with several physical and psychological problems in youth (Gunn et al., 2019). Previous studies have shown that poor sleep quality and sleep deprivation are observed in 31–53 % of adolescents (Li et al., 2020, Cavalcanti et al., 2021), being one of the most concerning in this age group. In addition to the time spent on screen time, sleep quality seems to be influenced by the level of physical activity (PA) (Cavalcanti et al., 2021).

PA increased sleep efficiency and duration regardless of the mode and intensity of activity in different populations (Dolezal et al., 2017). Despite the lack of consensus about the mechanisms behind the relationship between PA and sleep quality (Dolezal et al., 2017), PA contributes to the release of hormones such as endorphins and serotonin that promotes feelings of relaxation and may contribute to sleep quality. While screen time is not associated with PA levels (Dahlgren et al., 2021), screen time is negatively associated with moderate–vigorous PA in adolescents (Sanz-Martín et al., 2022).

Although PA improve sleep patterns and decrease screening time in adolescents (Christofaro et al., 2016), the potential influence of PA levels on the relationship between sleep quality/ duration and screen time remains unclear. Thus, we analyzed the association between sleep disorders and screen time according to PA levels in adolescents. We expected that the relationships between screen time and sleep quality may be attenuated in physically active adolescents.

## 2 Methods

### 2.1 Sample

The study was conducted in the Presidente Prudente city, Brazil. All procedures were approved by the local ethical committee. The inclusion criteria were: i) individuals aged 10–17 years; ii) and enrolled in primary or secondary schools. The exclusion criteria were: i) not meeting the recommendations for evaluation; ii) lack informed consent signed by the parents/guardian. The study met the institution’s guidelines for protection of human subjects concerning safety and privacy. The present study was approved by the Ethics and Research Committee of Universidade Estadual Paulista.

Among the 36 schools, one public school in each region was randomly selected, and all classrooms within these schools were invited to participate. As private schools are not present in all regions, two of them were selected at random from all private schools in the city. The sample size calculation indicated a minimum of 591 participants. Due to the lack of randomization of participants by classrooms, a design effect correction of 1.5 was applied and 10 % was added to anticipate potential sample losses, resulting in a final sample of 975 participants.

### 2.2 Sleep parameters

Mini Sleep Questionnaire was used to assess sleep quality (Zomer et al., 1985). Answers are given on a seven-point Likert scale: ranging from 1 (never) to 7 (always). The total sleep quality score ranges from 10 to 70 points and is categorized into four levels of difficulty sleeping: good quality (10–24 points); mild (25–27 points); moderate (28–30 points); and severe difficulty (≥31 points) (Zomer et al., 1985). For this study, the sample size was classified as “good sleep quality” (<25 points) and “poor sleep quality” (≥25 points). This instrument had its consistency and reliability tested on Brazilian undergraduate students, obtaining satisfactory values (Cronbach's alpha value = 0.770) (Falavigna et al., 2011).

The sleep duration was calculated by the difference between bedtime and the wake up time, self-reported. The cutoff point of eight hours/day of sleep was used to classify the sample as “sufficient sleep” (≥8 h) and “insufficient sleep” (<8 h).

### 2.3 Screen time

Screen time was assessed based on the number of hours of use on devices (i.e., TV, computer, video game and smartphone/tablet). Participants were asked about the number of hours per day they used this equipment during a typical weekday and typical weekend day (Hardy et al., 2007). This type of screen time assessment has been used in several epidemiological studies (Delfino et al., 2018, Mesquita et al., 2023, Tebar et al., 2023 Apr 8, Lemes et al., 2022 Jan, Veldman et al., 2023). The sample was classified into screen time levels by tertile, where adolescents located in the 3rd tertile were classified as “high screen time”, 2nd tertile as “moderate”, and those in ffirst tertile as “low screen time”.

### 2.4 Physical activity

The Baecke questionnaire (Baecke et al., 1982) was utilized to evaluate the habitual practice of PA across various domains, including school/work, sports/physical exercises during leisure time, and free time/locomotion related to occupation. The Baecke questionnaire had its reproducibility and validity tested in Brazilian pediatric populations and obtained good values (r = 0.55 to 0.82 and agreement ranging from 33.5 % to 76.6 %) (Guedes et al., 2006). Total score of the questionnaire was considered in the analysis. Adolescents located in the 4th quartile were considered as physically active, while the others classified as insufficiently physically active. This definition was adopted due to the lack of a cutoff point of Baecke questionnaire to define recommended levels of physical activity, which has a dimensionless score considering different intensities, duration and frequency, without an applicable metric (i.e. metabolic equivalent, counts or minutes). In this sense, the term insufficiently physically active was used to classify those participants who had any amount of physical activity that was not sufficient to be at the highest level, once it was not possible to infer about absence of physical activity practice to consider those participants as physically inactive.

### 2.5 Body mass index

For anthropometric assessment, adolescents were measured barefoot and wearing light clothing. Body weight was measured using a digital scale (Plenna®, São Paulo, Brazil) with 0.1 kg precision, and height was measured by a wall stadiometer, with 0.1 cm precision. The body mass index (BMI) was calculated by dividing body weight in kilograms by the square of height in meters (kg / m^2^).

### 2.6 Statistical analysis

The sample characteristics were presented as mean and standard deviation for continuous variables and in relative and absolute frequency for categorical variables. The difference in proportions of participants with poor sleep quality and with insufficient sleep according to screen time levels was analyzed by qui-square test in physically and insufficiently physically active adolescents. The association of sleep quality and sleep duration (dependent variables) with the screen time levels of adolescents was analyzed by logistic regression models adjusted by age and sex (model 1), and for age, sex, and BMI (model 2). These covariates were included due to be significantly correlated with the main variables of the study, being considered as potential confounders. This analysis was stratified by the PA level (physically and insufficiently active) of participants to investigate whether PA could be an effect modifier in the association between screen time and sleep. The software IBM SPSS version 25.0 was used for statistical analysis with significance level at p < 0.05 and 95 % confidence interval.

## 3 Results

The sample of the present study was composed of 1010 adolescents (556 girls). The prevalence of poor sleep quality was 52.3 %, while 46.6 % reported sleeping less than eight hours a day. When comparing the sample characterization variables between insufficiently active adolescents and physically active adolescents, there was no statistically significant difference for age, BMI, screen time, sleep time and sleep quality (Table 1).

**Table 1:** Characteristics of Brazilian adolescents participating in the study in 2014–2015 (n = 1010).

	**Insufficiently physically active (n = 754)**	**Physically active (n = 256)**	
	**Mean (SD)**	**Mean (SD)**	**P-value**
**Age (years)**	13.11 (2.41)	13.31 (2.20)	0.238
**BMI (kg/m^2^)**	20.29 (4.33)	20.61 (4.13)	0.309
**Sleep quality (score)**	25.72 (8.09)	25.48 (8.21)	0.678
**Sleep duration (hours/day)**	7.53 (1.68)	7.51 (1.59)	0.901
**Screen time (hours/day)**	10.46 (5.58)	10.94 (5.59)	0.237
**Physical activity (B. score)**	7.87 (1.81)	12.87 (1.29)	<0.001

BMI = Body mass index; B. score = Baecke’s score; SD = Standard deviation.

**Fig. 1:**
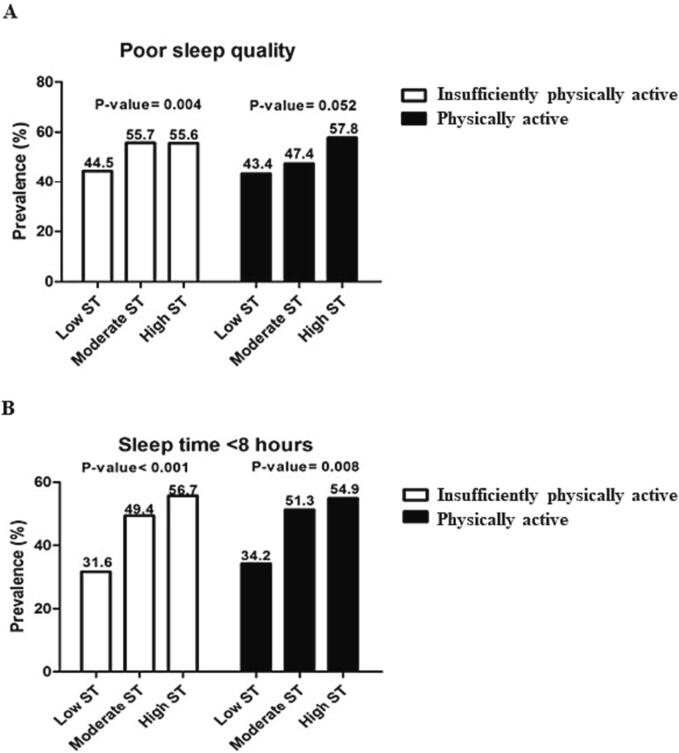
Prevalence of poor sleep quality and insufficient sleep time according screen time behaviors in inactive and physically active Brazilian adolescents in 2014–2015 (n = 1010).

The prevalence of poor sleep quality was higher in insufficiently physically active adolescents with high screen time (p = 0.004). The prevalence of sleeping < 8 h/day was also higher in adolescents with high screen time behaviors, and the differences were significant in both active (p = 0.008) and insufficiently physically active (p < 0.001) adolescents (Fig. 1).

Table 2 shows the associations between poor sleep quality and less than 8 sleeping hours with the level of screen time inactive and active adolescents. Two analysis models were created, the first model adjusted for sex and age and the second adjusted for BMI. In the first model, it is observed that high screen time behaviors were associated with poor sleep quality in inactive adolescents. In the second model, with BMI, a association was observed with high screen time and poor sleep quality and low sleep duration for inactive adolescents.

**Table 2:** Logistic binary regression analyzing the association of poor sleep quality and insufficient sleep time with screen time in Brazilian adolescents according to physical activity level in 2014–2015 (n = 1010).

	**Poor sleep quality**		**Insufficient sleep time (<8 h)**	
	**OR (95 %CI)**	**p-value**	**OR (95 %CI)**	**p-value**
***Insufficiently physically active***				
**Model 1**				
Low screen time	1.00		1.00	
Moderate screen time	1.38 (0.96–2.00)	0.087	1.29 (0.86–1.93)	0.214
High screen time	1.42 (0.98–2.08)	0.069	**1.57 (1.04**–**2.37)**	**0.032**
**Model 2**				
Low screen time	1.00		1.00	
Moderate screen time	1.41 (0.98–2.06)	0.069	1.22 (0.81–1.84)	0.332
High screen time	**1.45 (1.01**–**2.12)**	**0.049**	**1.52 (1.01**–**2.03)**	**0.046**
***Physically active***				
**Model 1**				
Low screen time	1.00		1.00	
Moderate screen time	1.19 (0.62–2.28)	0.583	1.66 (0.82–3.24)	0.162
High screen time	1.80 (0.97–3.37)	0.064	1.61 (0.84–3.09)	0.157
**Model 2**				
Low screen time	1.00		1.00	
Moderate screen time	1.18 (0.61–2.26)	0.611	1.68 (0.84–3.36)	0.141
High screen time	1.78 (0.94–3.32)	0.073	1.50 (0.77–2.91)	0.230

Model 1: adjusted by sex and age; Model 2: adjusted by sex, age, and BMI. OR = Odds ratio; CI = Confidence interval.

## 4 Discussion

Our study analyzed the association between sleep disorders and screen time according to PA levels in adolescents. When considering the proportions (Fig. 1), the prevalence of high screen time and poor sleep quality was significantly higher only in insufficiently active adolescents. However, when considering the inadequate sleep time, the high screen time was significantly higher both in insufficiently active adolescents and in those considered physically active. However, when considering multivariate analysis, we observed that adolescents with high screen time were approximately 50 % more likely to experience poor sleep quality and insufficient sleep duration, regardless of confounding factors. In physically active adolescents, this relationship was not observed.

Time spent on screen devices is negatively associated with sleep quality and duration in insufficiently physically active adolescents. The nocturnal habit of using screen devices or the presence of electronic devices in the living space has been strongly associated with insufficient sleep time in adolescents (Netsi et al., 2017). The brightness of the screen devices can inhibit melatonin production, leading to lighter and delayed sleep patterns. Furthermore, the interactive nature of screen devices and the stimulating content they present can impede both mental and physical relaxation, resulting in longer sleep onset times for adolescents (Hale and Guan, 2015, Hersh et al., 2015). Despite sleeping for more than 8 h, the presence of a late circadian rhythm (sleeping and waking up later) in these adolescents does not show a positive association with health (Vadnie and McClung, 2017, Shaw et al., 2016). Poor sleep quality and duration in adolescents have been linked to detrimental effects on cognitive function, mood, attention, behavior, and overall quality of life (Vadnie and McClung, 2017). PA is an essential strategy to improve sleep quality in adolescents (Dolezal et al., 2017).

Physical inactivity seems to be linked to the imbalance of the circadian cycle (Koren et al., 2016), leading to detrimental outcomes such as the disrupt reward-related brain function (Hasler et al., 2014). Conversely, promoting PA has demonstrated effectiveness in providing benefits directly related to improved sleep quality (Dolezal et al., 2017). Additionally, our study found no association between poor quality of sleep and excessive screen time in physically active adolescents. The increase in daily energy expenditure triggers a higher need for recovery and contributes to improvements in sleep quality (Vadnie and McClung, 2017). Moreover, PA leads to an increase in the production of substances associated with well-being (i.e., endorphin and serotonin), potentially promoting better sleep quality and mitigate the negative influence of excessive screen time in adolescents.

Despite the interesting results, our study has some limitations. One of them is the subjective assessment of sleep quality and time, not using more accurate methods such as polysomnography, for example. However, we highlight the difficulty and high cost of using this methodology in epidemiological studies with a large number of participants. Another point is the fact that physical activity was also assessed sub-actively, which may be inherent to memory biases. Despite these limitations, the random selection of a sample > 1000 participants and adjustment for potential confounding factors correspond to positive aspects of the study. Our study advances the existing knowledge by demonstrating that physically active practice can attenuate the influence of screen time on poor sleep quality in adolescents. Therefore, promoting PA programs in adolescents, regardless of sex and age, is essential to enhance health and improve quality of life.

## 5 Conclusion

High screen time was associated with poor sleep quality and duration specifically in insufficiently physically active adolescents. These results suggest that high PA levels may contribute to improving sleep patterns in adolescents.

## 6 Disclosure of ethical statements

The present study was approved by the Ethics and Research Committee of Universidade Estadual Paulista and all participants signed a consent form to participate in the study.

## 7 Disclosure of funding

The authors would like to thank the *Coordenação de Aperfeiçoamento de Pessoal de Nível Superior* (Capes) [Finance code 001] for financial support. DGDC and RMRD holds a Productivity Fellowship from the National Council for Scientific and Technological Development (Grant number: 305886/2022-3 and 308954/2021-1). The authors declare no competing interest.

## CRediT authorship contribution statement

**Amanda B. dos Santos:** Writing – original draft, Methodology, Data curation, Conceptualization. **Wagner L. Prado:** Writing – review & editing, Supervision, Investigation. **William R. Tebar:** Writing – original draft, Methodology, Formal analysis, Data curation, Conceptualization. **Jared Ingles:** Writing – review & editing. **Gerson Ferrari:** Writing – review & editing, Methodology, Investigation. **Priscila K. Morelhão:** Writing – review & editing. **Luan O. Borges:** Writing – original draft. **Raphael M. Ritti Dias:** Writing – review & editing, Investigation, Formal analysis. **Victor S. Beretta:** Writing – review & editing, Visualization, Methodology. **Diego G.D. Christofaro:** Data curation, Conceptualization, Methodology, Supervision, Formal analysis, Writing – review & editing.

## Declaration of competing interest

The authors declare that they have no known competing financial interests or personal relationships that could have appeared to influence the work reported in this paper.

## Data Availability

Data will be made available on request.
